# Supplementary material for: Associations between polygenic risk of coronary artery disease and type 2 diabetes, lifestyle, and cardiovascular mortality: A prospective UK Biobank study
Source: Front Cardiovasc Med. 2022 Aug 17;9:919374. doi: 10.3389/fcvm.2022.919374 (PMC9428483; doi:10.3389/fcvm.2022.919374)
Supplement: Supplementary file 1 [file Data_Sheet_1.pdf]

SUPPLEMENTARY MATERIAL

Associations between polygenic risk of coronary artery disease and type 2 diabetes, lifestyle, and cardiovascular mortality: A prospective UK Biobank study

Contents

Supplementary Table 1. Detailed definitions of lifestyle factors and lifestyle behavior.

Supplementary Table 2. Detailed definitions of comorbidities.

Supplementary Table 3. The number of cases with missing data for each variable.

Supplementary Table 4. Characteristics of participants at baseline according to categories of PRS for CAD and T2DM.

Supplementary Table 5. Hazard ratios and 95% confidential intervals for cardiovascular mortality using Cox proportional regression model.

Supplementary Table 6. Sensitivity analysis for atherosclerotic cardiovascular mortality.

Supplementary Table 7. Sensitivity analysis for mortality related to CAD.

Supplementary Table 8. Sensitivity analysis after excluding participants who had chronic liver disease, chronic lung disease, chronic kidney disease, or cancer at baseline.

Supplementary Table 9. Hazard ratios and 95% confidential intervals for cardiovascular mortality according to genetic risk for CAD and lifestyle behaviors.

Supplementary Table 10. Hazard ratios and 95% confidential intervals for cardiovascular mortality according to genetic risk for T2DM and lifestyle behaviors.

Supplementary Table 11. Hazard ratios and 95% confidential intervals for cardiovascular mortality according to genetic risk and age category.

Supplementary Table 12. Hazard ratios and 95% confidential intervals for cardiovascular mortality according to genetic risk and sex category.

Supplementary Table 13. Hazard ratios and 95% confidential intervals for cardiovascular mortality according to genetic risk and prevalent disease of T2DM or CAD at baseline.

Supplementary Figure 1. Density and prevalence plot according to the PRS distribution (A) CAD and (B) T2DM.

Supplementary Figure 2. 10-year cardiovascular mortality rates according to (A) genetic risk for CAD and (B) genetic risk for T2DM and lifestyle behavior.

Supplementary Figure 3. Forest plot of cardiovascular mortality according to genetic risk for CAD and four lifestyle behaviors.

Supplementary Figure 4. Forest plot of cardiovascular mortality according to genetic risk for T2DM and four lifestyle behaviors.

Supplementary Figure 5. 10-year cardiovascular mortality rates according to (A) genetic risk for CAD and age categories, (B) genetic risk for T2DM and age categories, (C) genetic risk for CAD and sex categories, and (D) genetic risk for T2DM and sex categories.

Supplementary Figure 6. 10-year cardiovascular mortality rates according to genetic risk and prevalent disease status; (A) genetic risk for CAD and CAD history at baseline, (B) genetic risk for CAD and T2DM history at baseline, (C) genetic risk for T2DM and CAD history at baseline, and (D) genetic risk for T2DM and T2DM history at baseline.

Supplementary Figure 7. Forest plot of cardiovascular mortality according to genetic risk and sex in patients with diabetes at baseline.

Supplementary Table 1. Detailed definitions of lifestyle factors and lifestyle behavior.

Lifestyle factors	Component	Healthy lifestyle	Field ID of UK biobank
Current smoking	Current smoking at baseline	Absence	20116
Obesity	BMI at baseline	<30 kg/m^2	21001
Physical activity	Number of days per week of physical activity 10+ minutes	Participating in moderate activity ≥5 days a week or vigorous activity ≥3 days a week	884 (Moderate physical activity 10+ minutes)<br>904 (Vigorous physical activity 10+ minutes)
Eating habits	At least half of all following diet components was considered as a healthy lifestyle, less than half was considered as an unhealthy lifestyle		
	Fruit	≥3 serving/day	1309 (Fresh fruit)<br>1319 (Dried fruit)
	Vegetable	≥3 serving/day	1289 (Cooked vegetables)<br>1299 (Salad or raw vegetables)
	Whole grains	≥3 serving/day	1438, 1448 (Wholemeal or wholegrain bread)<br>1458, 1468 (Bran, oat, muesli cereal)
	Fish	≥2 serving/week	1329 (Oily fish)<br>1339 (Non-oily fish)
	Dairy	≥2.5 serving/week	1408 (Cheese)<br>1418 (Milk)
	Refined grains	≤1.5 serving/week	1438, 1448 (Wholemeal or wholegrain bread)<br>1458, 1468 (Bran, oat, muesli cereal)
	Processed meats	≤1 serving/week	1349 (Processed meat)<br>3680 (Age when last ate any kind of meat, 0 if indicated having never eaten meat)
	Unprocessed meats	≤1.5 serving/week	1359 (Poultry)<br>1369 (Beef)<br>1379 (Lamb)<br>1389 (Pork)<br>3680 (Age when last ate any kind of meat, 0 if indicated having never eaten meat)
	Sugar-sweetened beverages	≤1 serving/week	6144 (Never eats sugar or foods/drinks containing sugar)
Lifestyle behavior	Favorable	Having at least three healthy lifestyle factors	
	Intermediate	Having two healthy lifestyle factors	
	Unfavorable	Having one or fewer healthy lifestyle factor	

Supplementary Table 2. Detailed definitions of comorbidities.

Disease	Path	Field ID	Code
Dyslipidemia	Verbal interview	Non-cancer illness, self-report (20002)	High cholesterol (1473)
	First occurrence before enrollment	First reported of disorders of lipoprotein metabolism and other lipidemia (130815, 130816)	E78.x
	Medication	Medication for cholesterol, blood pressure or diabetes (6177)	Cholesterol lowering medication
Hypertension	Verbal interview	Non-cancer illness, self-report (20002)	1065, 1072
	Touchscreen	Vascular/heart problems diagnosed by doctor (6150)	High blood pressure
	First occurrence before enrollment	First reported of essential hypertension (131286, 131287)	I10.x
		First reported of hypertensive heart disease (131288, 131289)	I11.x
		First reported of hypertensive renal disease (131290, 131291)	I12.x
		First reported of hypertensive heart and renal disease (131292, 131293)	I13.x
		First reported of secondary hypertension (131294, 131295)	I15.x
	Medication	Medication for cholesterol, blood pressure or diabetes (6177)	Blood pressure medication
Type 2 diabetes mellitus	Verbal interview	Non-cancer illness, self-report (20002)	Diabetes (1220)<br>Type 2 diabetes (1223)
	Touchscreen	Diabetes diagnosed by doctor (2443)	Yes
	First occurrence before enrollment	First reported of non-insulin-dependent diabetes mellitus (130708, 130709)	E11.x
		First reported of unspecified diabetes mellitus (130714, 130715)	E14.x
	Medication	Treatment/medication code (20003)	Insulin (1140883066)<br>Metformin (1140884600, 1141189090)<br>Sulfonylurea (1141152590, 1140874744, 1140874718, 1141156984)<br>Acarbose (1140868902)<br>Thiazolidinedione (1141171646)<br>Meglitinide (1141168660, 1141173882)
	HbA1c at baseline	Glycated hemoglobin (HbA1c) (30750)	≥6.5%
	Verbal interview for exclusion type 1 diabetes	Non-cancer illness, self-report (20002)	Type 1 diabetes (1222)
	First occurrence for exclusion type 1 diabetes	First reported of insulin-dependent diabetes mellitus (130706, 130707)	E10.x
Coronary artery disease	Verbal interview	Non-cancer illness, self-report (20002)	1074 (angina)<br>1075 (heart attack/myocardial infarction)
	Touchscreen	Vascular/heart problems diagnosed by doctor (6150)	Heart attack, angina
	First occurrence before enrollment	First reported of angina pectoris (131296, 131297)	I20.x
		First reported of acute myocardial infarction (131298, 131299)	I21.x
		First reported of subsequent myocardial infarction (131300, 131301)	I22.x
		First reported of certain current complications following acute myocardial infarction (131302, 131303)	I23.x
		First reported of other acute ischemic heart diseases (131304, 131305)	I24.x
		First reported of chronic ischemic heart disease (131306, 131307)	I25.x
Heart failure	Verbal interview	Non-cancer illness, self-report (20002)	Heart failure/pulmonary edema (1076)<br>Cardiomyopathy (1079)
	First occurrence before enrollment	First reported of heart failure (131354, 131355)	I50.x
	Hospital inpatient	Hospital inpatient record, diagnosis ICD10 code (41270)	I11, I110, I113, I132, I255, I420, I421, I422, I425, I428, I50, I500, I501, IO509
		Hospital inpatient record, diagnosis ICD9 code (41271)	4254, 4280, 4281, 4289
Ischemic stroke	Verbal interview	Non-cancer illness, self-report (20002)	Stroke (1081)<br>Ischemic stroke (1583)
	Touchscreen	Vascular/heart problems diagnosed by doctor (6150)	Stroke
	First occurrence before enrollment	First reported of cerebral infarction (131366, 131367)	I63.x
		First reported of stroke, not specified as hemorrhage or infarction (131368, 131369)	I64.x

Chronic lung disease	Verbal interview	Non-cancer illness, self-report (20002)	Asthma (1111)<br>Chronic obstructive airway disease/COPD (1112)<br>Emphysema/chronic bronchitis (1113)<br>Bronchiectasis (1114)<br>Interstitial lung disease (1115)
	First occurrence before enrollment	First reported of bronchitis, not specified as acute or chronic (131484, 131485)	J40.x
		First reported of simple and mucopurulent chronic bronchitis (131486, 131487)	J41.x
		First reported of unspecified chronic bronchitis (131488, 131489)	J42.x
		First reported of emphysema (131490, 131491)	J43.x
		First reported of other chronic obstructive pulmonary disease (131492, 131493)	J44.x
		First reported of asthma (131494, 131495)	J45.x
		First reported of status asthmaticus (131496, 131497)	J46.x
		First reported of bronchiectasis (131498, 131499)	J47.x
		First reported of coalworker's pneumoconiosis (131500, 131501)	J60.x
		First reported of pneumoconiosis due to asbestos and other mineral fibers (131502, 131503)	J61.x
		First reported of pneumoconiosis due to dust containing silica (131504, 131505)	J62.x
		First reported of pneumoconiosis due to other inorganic dusts (131506, 131507)	J63.x
		First reported of unspecified pneumoconiosis (131508, 131509)	J64.x
		First reported of airway disease due to specific organic dust (131512, 131513)	J66.x
		First reported of hypersensitivity pneumonitis due to organic dust (131514, 131515)	J67.x
Chronic liver disease	Verbal interview	Non-cancer illness, self-report (20002)	Hepatitis (1155)<br>Infective/viral hepatitis (1156)<br>Non-infective hepatitis (1157)<br>Liver failure/cirrhosis (1158)
	First occurrence before enrollment	First reported of chronic viral hepatitis (130200, 130201)	B18.x
		First reported of esophageal varices (131406, 131407)	I85.x
		First reported of alcoholic liver disease (131658, 131659)	K70.x
		First reported of toxic liver disease (131660, 131661)	K71.x
		First reported of hepatic failure, not elsewhere classified (131662, 131663)	K72.x
		First reported of chronic hepatitis, not elsewhere classified (131664, 131665)	K73.x
		First reported of other diseases of liver (131670, 131671)	K76.x
Chronic kidney disease	Verbal interview	Non-cancer illness, self-report (20002)	Renal/kidney failure (1192)<br>Renal failure requiring dialysis (1193)<br>Renal failure not requiring dialysis (1194)
	First occurrence before enrollment	First reported of chronic renal failure (132030, 132031)	N18.x
		First reported of unspecified renal failure (132032, 132033)	N19.x
Cancer	Verbal interview	Cancer, self-report (20001)	1001-1012, 1015-1048, 1050-1053, 1055,1056, 1058-1068, 1070-1082, 1084-1088
	First occurrence before enrollment	Cancer register (40006)	C00-C97

Supplementary Table 3. The number of cases with missing data for each variable.

Variable	n (%)
Total	377,909
Systolic blood pressure	341 (0.1)
Diastolic blood pressure	339 (0.1)
Height	807 (0.2)
Weight	1,058 (0.3)
Body mass index	1,192 (0.3)
Waist circumference	626 (0.2)
Current smoking	1331 (0.4)
Alcohol frequency	336 (0.1)
Physical activity	1,686 (0.4)
Lifestyle behavior	14,431 (3.8)
Total cholesterol	17,588 (4.7)
Triglyceride	17,880 (4.7)
HDL-cholesterol	48,177 (12.7)
LDL-cholesterol	18,257 (4.8)
Creatinine	17,772 (4.7)
C-reactive protein	18,382 (4.9)
AST	18,914 (5.0)
ALT	17,721 (4.7)
Glucose	48,393 (12.8)
HbA1c	17,768 (4.7)
Lipoprotein(a)	91,389 (24.2)
White blood cell	11,203 (3.0)
Hemoglobin	11,199 (3.0)
Platelet	11,202 (3.0)
Albumin	48,029 (12.7)
Total bilirubin	19,094 (5.1)

AST, Aspartate aminotransferase; ALT, Alanine aminotransferase.

Supplementary Table 4. Characteristics of participants at baseline according to categories of PRS for CAD and T2DM.

	Total	PRS for CAD					PRS for T2DM				
		**Low** 0th-19th percentile	**Intermediate** 20th-79th percentile	**High** 80th-98th percentile	**Very high** 99th percentile	***P* value**	**Low** 0th-19th percentile	**Intermediate** 20th-79th percentile	**High** 80th-98th percentile	**Very high** 99th percentile	***P* value**
	**(n=377,909)**	**(n=75,589)**	**(n=226,767)**	**(n=71,776)**	**(n=3,777)**		**(n=75,607)**	**(n=226,783)**	**(n=71,744)**	**(n=3,775)**	
**Demographics & Physical measurement**											
Age (years)	56.5 ± 7.9	56.5 ± 7.9	56.5 ± 8.0	56.4 ± 7.9	56.3 ± 7.9	0.002	56.5 ± 7.9	56.5 ± 8.0	56.5 ± 7.9	56.3 ± 7.9	0.05
Men	174,829 (46.3)	35,020 (46.3)	104,958 (46.3)	33,111 (46.1)	1,740 (46.1)	0.86	34,867 (46.1)	104,954 (46.3)	33,238 (46.3)	1,770 (46.9)	0.71
Systolic blood pressure (mmHg)	140.3 ± 19.7	139.7 ± 19.5	140.3 ± 19.7	140.7 ± 19.7	141.2 ± 19.7	<0.001	139.5 ± 19.7	140.3 ± 19.6	141.1 ± 19.6	142.3 ± 19.4	<0.001
Diastolic blood pressure (mmHg)	82.3 ± 10.7	82.2 ± 10.6	82.3 ± 10.7	82.4 ± 10.7	82.6 ± 10.8	0.005	81.9 ± 10.6	82.3 ± 10.7	82.7 ± 10.6	83.5 ± 10.6	<0.001
BMI (kg/m^2)	27.4 ± 4.8	27.3 ± 4.7	27.4 ± 4.7	27.5 ± 4.8	27.7 ± 4.9	<0.001	27.0 ± 4.6	27.4 ± 4.8	27.8 ± 4.9	28.5 ± 5.1	<0.001
Waist circumference (cm)	90.4 ± 13.5	90.2 ± 13.5	90.4 ± 13.5	90.5 ± 13.6	90.9 ± 13.7	<0.001	89.3 ± 13.3	90.4 ± 13.5	91.5 ± 13.6	93.1 ± 13.7	<0.001
Lifestyle behavior						<0.001					<0.001
Favorable	196,450 (54.0)	39,836 (54.8)	117,694 (54.0)	36,999 (53.5)	1,921 (52.6)		40,929 (56.2)	117,806 (54.0)	35,957 (52.1)	1,758 (48.8)	
Intermediate	122,695 (33.8)	24,211 (33.3)	73,788 (33.9)	23,431 (33.9)	1,265 (34.6)		24,001 (33.0)	73,666 (33.8)	23,718 (34.4)	1,310 (36.3)	
Unfavorable	44,333 (12.2)	8,697 (12.0)	26,495 (12.2)	8,675 (12.6)	466 (12.8)		7,855 (10.8)	26,649 (12.2)	9,291 (13.5)	538 (14.9)	
**Laboratory findings**											
HbA1c (%)	5.4 ± 0.6	5.4 ± 0.6	5.4 ± 0.6	5.5 ± 0.6	5.5 ± 0.6	<0.001	5.4 ± 0.5	5.4 ± 0.6	5.5 ± 0.7	5.6 ± 0.8	<0.001
Estimated GFR (ml/min/1.73m^2)	78.6 ± 14.2	78.6 ± 14.1	78.5 ± 14.3	78.6 ± 14.3	78.4 ± 14.4	0.95	78.5 ± 14.0	78.5 ± 14.2	78.7 ± 14.4	79.0 ± 14.9	<0.001
Total cholesterol (mmol/L)	5.72 ± 1.15	5.69 ± 1.12	5.72 ± 1.15	5.74 ± 1.17	5.76 ± 1.23	<0.001	5.73 ± 1.12	5.72 ± 1.15	5.70 ± 1.17	5.69 ± 1.22	<0.001
Triglyceride (mmol/L)	1.76 ± 1.02	1.75 ± 1.01	1.76 ± 1.02	1.76 ± 1.03	1.76 ± 1.08	0.08	1.68 ± 0.97	1.76 ± 1.02	1.84 ± 1.07	1.94 ± 1.14	<0.001
HDL-cholesterol (mmol/L)	1.45 ± 0.38	1.46 ± 0.38	1.45 ± 0.38	1.44 ± 0.38	1.43 ± 0.38	<0.001	1.48 ± 0.38	1.45 ± 0.38	1.42 ± 0.38	1.39 ± 0.38	<0.001
LDL-cholesterol (mmol/L)	3.57 ± 0.87	3.54 ± 0.85	3.57 ± 0.87	3.60 ± 0.89	3.63 ± 0.93	<0.001	3.57 ± 0.85	3.57 ± 0.87	3.57 ± 0.89	3.57 ± 0.92	0.11
**Baseline major comorbidity**											
Coronary artery disease	21,678 (5.7)	2,950 (3.9)	12,592 (5.6)	5,639 (7.9)	497 (13.2)	<0.001	3,675 (4.9)	13,183 (5.8)	4,529 (6.3)	291 (7.7)	<0.001
Type 2 diabetes mellitus	15,034 (4.2)	2,725 (3.8)	9,030 (4.2)	3,099 (4.6)	180 (5.1)	<0.001	1,758 (2.5)	8,724 (4.1)	4,222 (6.3)	330 (9.4)	<0.001
Dyslipidemia	69,767 (18.5)	11,771 (15.6)	41,575 (18.3)	15,300 (21.3)	1,121 (29.7)	<0.001	12,222 (16.2)	41,816 (18.4)	14,847 (20.7)	882 (23.4)	<0.001
Hypertension	112,350 (29.7)	20,962 (27.7)	67,299 (29.7)	22,733 (31.7)	1,356 (35.9)	<0.001	20,087 (26.6)	67,451 (29.7)	23,404 (32.6)	1,408 (37.3)	<0.001
Myocardial infarction	9,472 (2.5)	1,116 (1.5)	5,391 (2.4)	2,705 (3.8)	260 (6.9)	<0.001	1,510 (2.0)	5,822 (2.6)	2,007 (2.8)	133 (3.5)	<0.001
Heart failure	2,626 (0.7)	410 (0.5)	1,530 (0.7)	632 (0.9)	54 (1.4)	<0.001	442 (0.6)	1,607 (0.7)	547 (0.8)	30 (0.8)	<0.001
Ischemic stroke	6,744 (1.8)	1,206 (1.6)	4,107 (1.8)	1,355 (1.9)	76 (2.0)	<0.001	1,225 (1.6)	4,030 (1.8)	1,399 (1.9)	90 (2.4)	<0.001
Chronic lung disease	55,410 (14.7)	11,150 (14.8)	33,251 (14.7)	10,471 (14.6)	538 (14.2)	0.73	10,422 (13.8)	33,467 (14.8)	10,931 (15.2)	590 (15.6)	<0.001
Chronic kidney disease	5,766 (1.5)	1,050 (1.4)	3,506 (1.5)	1,138 (1.6)	72 (1.9)	0.002	1,023 (1.4)	3,492 (1.5)	1,174 (1.6)	77 (2.0)	<0.001
Cancer	45,102 (11.9)	9,318 (12.3)	26,988 (11.9)	8,393 (11.7)	403 (10.7)	<0.001	9,079 (12.0)	27,007 (11.9)	8,572 (11.9)	444 (11.8)	0.88
Charlson comorbidity index	0.57 ± 0.97	0.55 ± 0.95	0.57 ± 0.97	0.59 ± 0.99	0.60 ± 0.99	<0.001	0.53 ± 0.94	0.57 ± 0.97	0.61 ± 1.00	0.66 ± 1.04	<0.001

Data are n (%) or mean (SD). PRS, polygenic risk score; CAD, coronary artery disease; T2DM, type 2 diabetes mellitus; GFR, glomerular filtration rate.

Supplementary Table 5. Hazard ratios and 95% confidential intervals for cardiovascular mortality using Cox proportional regression model.

		No. of Events/ Total No.	Incidence/ 1000 person-year (95% CI)	Absolute risk (%)	Cardiovascular mortality													
					Crude		Model 1		Model 2		Model 3		Model 4		Model 5		Model 6	
					HR (95% CI)	*P* value	HR (95% CI)	*P* value	HR (95% CI)	*P* value	HR (95% CI)	*P* value	HR (95% CI)	*P* value	HR (95% CI)	*P* value	HR (95% CI)	*P* value
**CAD PRS**	Low risk	516/75589	0.77 (0.70–0.84)	0.68	Ref		Ref		Ref		Ref		Ref		Ref		Ref	
	Intermediate risk	1906/226767	0.94 (0.90–0.99)	0.84	1.23 (1.12–1.36)	<0.001	1.23 (1.12–1.36)	<0.001	1.19 (1.05–1.34)	0.004	1.24 (1.06–1.44)	0.005	1.17 (1.01–1.36)	0.04	1.17 (1.01–1.36)	0.04	1.16 (0.99–1.35)	0.05
	High risk	721/71776	1.12 (1.05–1.21)	1.00	1.47 (1.32–1.65)	<0.001	1.50 (1.33–1.68)	<0.001	1.49 (1.30–1.71)	<0.001	1.54 (1.29–1.83)	<0.001	1.38 (1.16–1.65)	<0.001	1.39 (1.16–1.65)	<0.001	1.37 (1.15–1.63)	<0.001
	Very high risk	67/3777	2.00 (1.55–2.54)	1.77	2.61 (2.02–3.36)	<0.001	2.68 (2.08–3.46)	<0.001	2.84 (2.10–3.84)	<0.001	2.24 (1.40–3.59)	<0.001	1.80 (1.12–2.90)	0.01	1.96 (1.22–3.14)	0.005	1.91 (1.19–3.07)	0.007
**T2DM PRS**	Low risk	554/75607	0.82 (0.75–0.89)	0.73	Ref		Ref		Ref		Ref		Ref		Ref		Ref	
	Intermediate risk	1916/226783	0.95 (0.91–0.99)	0.84	1.16 (1.05–1.27)	0.003	1.17 (1.07–1.29)	<0.001	1.16 (1.03–1.30)	0.01	1.24 (1.07–1.44)	0.004	1.20 (1.03–1.39)	0.02	1.17 (1.01–1.36)	0.04	1.17 (1.01–1.36)	0.04
	High risk	683/71744	1.07 (0.99–1.15)	0.95	1.30 (1.17–1.46)	<0.001	1.33 (1.19–1.49)	<0.001	1.26 (1.10–1.45)	<0.001	1.39 (1.16–1.65)	<0.001	1.32 (1.11–1.58)	0.002	1.26 (1.06–1.50)	0.01	1.26 (1.06–1.50)	0.01
	Very high risk	57/3775	1.70 (1.29–2.21)	1.51	2.08 (1.58–2.73)	<0.001	2.16 (1.64–2.83)	<0.001	2.09 (1.51–2.90)	<0.001	2.16 (1.43–3.27)	<0.001	2.03 (1.34–3.07)	<0.001	1.83 (1.21–2.78)	0.004	1.82 (1.20–2.76)	0.005

Model 1: Age + sex + genotyping array + first ten PC

Model 2: Model 1 + systolic BP + diastolic BP + BMI + waist circumference + current smoking + alcohol frequency + physical activity + eating habit

Model 3: Model 2 + HbA1c + total cholesterol + triglyceride + HDL cholesterol + LDL cholesterol + lipoprotein (a) + estimated GFR + AST + ALT + Total bilirubin + albumin + white blood cell + hemoglobin + platelet + C-reactive protein

Model 4: Model 3 + PRS-specific disease

Model 5: Model 3 + hypertension + dyslipidemia + heart failure + cancer + chronic kidney disease + stroke + chronic lung disease + chronic liver disease + Charlson comorbidity index

Model 6: Model 4 + aspirin + lipid-lowering agent + anti-hypertensive agent

CAD, coronary artery disease; PRS polygenic risk score; T2DM, type 2 diabetes; PC, principal component; BP, blood pressure; GFR, glomerular filtration rate; AST, Aspartate aminotransferase; ALT, Alanine aminotransferase; HR, hazard ratio; CI, confidence interval.

CAD PRS and T2DM PRS of Model 4 were adjusted for CAD and T2DM, respectively.

Supplementary Table 6. Sensitivity analysis for atherosclerotic cardiovascular mortality.

					Cardiovascular mortality													
		No. of Events/ Total No.	Incidence/ 1000 person-year (95% CI)	Absolute risk (%)	Crude		Model 1		Model 2		Model 3		Model 4		Model 5		Model 6	
					HR (95% CI)	*P* value	HR (95% CI)	*P* value	HR (95% CI)	*P* value	HR (95% CI)	*P* value	HR (95% CI)	*P* value	HR (95% CI)	*P* value	HR (95% CI)	*P* value
CAD PRS	Low risk	269/75589	0.40 (0.35-0.45)	0.36	Ref		Ref		Ref		Ref		Ref		Ref		Ref	
	Intermediate risk	1165/226767	0.58 (0.54-0.61)	0.51	1.44 (1.27-1.65)	<0.0001	1.45 (1.27-1.65)	<0.0001	1.36 (1.16-1.59)	0.0001	1.34 (1.10-1.63)	0.0039	1.31 (1.07-1.60)	0.0083	1.25 (1.02-1.52)	0.0301	1.23 (1.01-1.50)	0.0382
	High risk	492/71776	0.77 (0.70-0.84)	0.69	1.93 (1.66-2.24)	<0.0001	1.96 (1.69-2.27)	<0.0001	1.92 (1.61-2.30)	<0.0001	1.92 (1.54-2.40)	<0.0001	1.86 (1.49-2.33)	<0.0001	1.69 (1.36-2.12)	<0.0001	1.66 (1.33-2.08)	<0.0001
	Very high risk	52/3777	1.55 (1.16-2.03)	1.38	3.88 (2.88-5.22)	<0.0001	3.99 (2.97-5.37)	<0.0001	4.11 92.90-5.83)	<0.0001	2.98 (1.73-5.13)	<0.0001	2.87 (1.64-5.01)	0.000224	2.56 (1.49-4.41)	0.000695	2.48 (1.44-4.27)	0.0011
T2DM PRS	Low risk	298/75607	0.44 (0.39-0.49)	0.39	Ref		Ref		Ref		Ref		Ref		Ref		Ref	
	Intermediate risk	1195/226783	0.59 (0.56-0.63)	0.53	1.34 (1.18-1.52)	<0.0001	1.36 (1.20-1.55)	<0.0001	1.41 (1.20-1.64)	<0.0001	1.53 (1.25-1.88)	<0.0001	1.46 (1.19-1.80)	0.0002	1.42 (1.16-1.73)	0.0007	1.42 (1.16-1.73)	0.0007
	High risk	446/71744	0.70 (0.63-0.77)	0.62	1.58 (1.37-1.83)	<0.0001	1.63 (1.40-1.88)	<0.0001	1.55 (1.30-1.86)	<0.0001	1.83 (1.45-2.30)	<0.0001	1.71 (1.35-2.15)	<0.0001	1.63 (1.29-2.05)	<0.0001	1.62 (1.29-2.05)	<0.0001
	Very high risk	39/3775	1.16 (0.83-1.59)	1.03	2.64 (1.89-3.68)	<0.0001	2.75 (1.97-3.84)	<0.0001	2.69 (1.81-4.00)	<0.0001	3.12 (1.92-5.07)	<0.0001	2.88 (1.77-4.69)	<0.0001	2.49 (1.53-4.07)	0.0003	2.49 (1.52-4.05)	0.0003

Model 1: Age + sex + genotyping array + first ten PC

Model 2: Model 1 + systolic BP + diastolic BP + BMI + waist circumference + current smoking + alcohol frequency + physical activity + eating habit

Model 3: Model 2 + HbA1c + total cholesterol + triglyceride + HDL cholesterol + LDL cholesterol + lipoprotein (a) + estimated GFR + AST + ALT + Total bilirubin + albumin + white blood cell + hemoglobin + platelet + C-reactive protein

Model 4: Model 3 + PRS-specific disease

Model 5: Model 3 + hypertension + dyslipidemia + heart failure + cancer + chronic kidney disease + stroke + chronic lung disease + chronic liver disease + Charlson comorbidity index

Model 6: Model 4 + aspirin + lipid-lowering agent + anti-hypertensive agent

CAD, coronary artery disease; PRS polygenic risk score; T2DM, type 2 diabetes; PC, principal component; BP, blood pressure; GFR, glomerular filtration rate; AST, Aspartate aminotransferase; ALT, Alanine aminotransferase; HR, hazard ratio; CI, confidence interval.

CAD PRS and T2DM PRS of Model 4 were adjusted for CAD and T2DM, respectively.

Supplementary Table 7. Sensitivity analysis for mortality related to CAD.

		No. of Events/ Total No.	Incidence/ 1000 person-year (95% CI)	Absolute risk (%)	Cardiovascular mortality													
					Crude		Model 1		Model 2		Model 3		Model 4		Model 5		Model 6	
					HR (95% CI)	*P* value	HR (95% CI)	*P* value	HR (95% CI)	*P* value	HR (95% CI)	*P* value	HR (95% CI)	*P* value	HR (95% CI)	*P* value	HR (95% CI)	*P* value
**CAD PRS**	Low risk	223/75589	0.33 (0.29-3.77)	0.30	Ref		Ref		Ref		Ref		Ref		Ref		Ref	
	Intermediate risk	1010/226767	0.50 (0.47-0.53)	0.45	1.51 (1.31-1.75)	<0.0001	1.51 (1.31-1.75)	<0.0001	1.41 (1.19-1.67)	<0.0001	1.36 (1.10-1.68)	0.0052	1.32 (1.07-1.64)	0.0111	1.26 (1.02-1.56)	0.3525	1.24 (1.00-1.54)	0.0480
	High risk	450/71776	0.70 (0.64-0.77)	0.63	2.13 (1.81-2.50)	<0.0001	2.16 (1.84-2.53)	<0.0001	2.09 (1.73-2.53)	<0.0001	2.05 (1.62-2.60)	<0.0001	1.98 (1.55-2.52)	<0.0001	1.80 (1.41-2.28)	<0.0001	1.75 (1.38-2.22)	<0.0001
	Very high risk	47/3777	1.40 (1.03-1.86)	1.24	4.23 (3.09-5.79)	<0.0001	4.34 (3.17-5.95)	<0.0001	4.47 (3.10-6.44)	<0.0001	3.16 (1.80-5.56)	<0.0001	3.04 (1.69-5.44)	0.0002	2.67 (1.52-4.71)	0.0007	2.58 (1.46-4.54)	0.0011
**T2DM PRS**	Low risk	267/75607	0.40 (0.35-0.45)	0.35	Ref		Ref		Ref		Ref		Ref		Ref		Ref	
	Intermediate risk	1023/226783	0.51 (0.48-0.54)	0.45	1.28 (1.12-1.46)	0.0003	1.30 (1.14-1.49)	0.0001	1.32 (1.12-1.56)	0.0008	1.48 (1.19-1.83)	0.0004	1.40 (1.13-1.75)	0.0021	1.36 (1.09-1.68)	0.0054	1.36 (1.09-1.68)	0.0054
	High risk	405/71744	0.63 (0.57-0.70)	0.57	1.60 (1.37-1.87)	<0.0001	1.64 (1.41-1.92)	<0.0001	1.53 (1.26-1.85)	<0.0001	1.86 (1.46-2.37)	<0.0001	1.72 (1.35-2.20)	<0.0001	1.65 (1.29-2.10)	<0.0001	1.64 (1.29-2.09)	<0.0001
	Very high risk	35/3775	1.04 (0.73-1.45)	0.93	2.64 (1.86-3.76)	<0.0001	2.73 (1.92-3.89)	<0.0001	2.45 (1.60-3.75)	<0.0001	2.85 (1.68-4.84)	0.000101	2.63 (1.55-4.47)	0.0035	2.24 (1.32-3.81)	0.0029	2.23 (1.31-3.79)	0.0030

Model 1: Age + sex + genotyping array + first ten PC

Model 2: Model 1 + systolic BP + diastolic BP + BMI + waist circumference + current smoking + alcohol frequency + physical activity + eating habit

Model 3: Model 2 + HbA1c + total cholesterol + triglyceride + HDL cholesterol + LDL cholesterol + lipoprotein (a) + estimated GFR + AST + ALT + Total bilirubin + albumin + white blood cell + hemoglobin + platelet + C-reactive protein

Model 4: Model 3 + PRS-specific disease

Model 5: Model 3 + hypertension + dyslipidemia + heart failure + cancer + chronic kidney disease + stroke + chronic lung disease + chronic liver disease + Charlson comorbidity index

Model 6: Model 4 + aspirin + lipid-lowering agent + anti-hypertensive agent

CAD, coronary artery disease; PRS polygenic risk score; T2DM, type 2 diabetes; PC, principal component; BP, blood pressure; GFR, glomerular filtration rate; AST, Aspartate aminotransferase; ALT, Alanine aminotransferase; HR, hazard ratio; CI, confidence interval.

CAD PRS and T2DM PRS of Model 4 were adjusted for CAD and T2DM, respectively.

Supplementary Table 8. Sensitivity analysis after excluding participants who had chronic liver disease, chronic lung disease, chronic kidney disease, or cancer at baseline.

					Cardiovascular mortality													
		No. of Events/ Total No.	Incidence/ 1000 person-year (95% CI)	Absolute risk (%)	Crude		Model 1		Model 2		Model 3		Model 4		Model 5		Model 6	
					HR (95% CI)	*P* value	HR (95% CI)	*P* value	HR (95% CI)	*P* value	HR (95% CI)	*P* value	HR (95% CI)	*P* value	HR (95% CI)	*P* value	HR (95% CI)	*P* value
**CAD PRS**	Low risk	317/55358	0.64 (0.57-0.72)	0.57	Ref		Ref		Ref		Ref		Ref		Ref		Ref	
	Intermediate risk	1201/166772	0.81 (0.76-0.85)	0.72	1.26 (1.11-1.42)	0.0003	1.26 (1.11-1.42)	0.0003	1.23 (1.06-1.43)	0.0059	1.30 (1.08-1.56)	0.0062	1.27 (1.05-1.53)	0.0134	1.25 (1.04-1.51)	0.0185	1.24 (1.04-1.50)	0.0202
	High risk	473/52973	1.00 (0.91-1.10)	0.89	1.56 (1.35-1.80)	<0.0001	1.58 (1.37-1.82)	<0.0001	1.54 (1.30-1.82)	<0.0001	1.63 (1.31-2.02)	<0.0001	1.60 (1.29-1.98)	<0.0001	1.51 (1.21-1.87)	0.0002	1.49 (1.20-1.85)	0.0003
	Very high risk	47/2806	1.88 (1.38-2.50)	1.68	2.93 (2.16-3.98)	<0.0001	2.97 (2.18-4.03)	<0.0001	3.32 (2.34-4.70)	<0.0001	2.35 (1.32-4.16)	0.0036	2.21 (1.22-4.00)	0.0090	2.11 (1.19-3.75)	0.0106	2.08 (1.17-3.70)	0.0122
**T2DM PRS**	Low risk	354/56236	0.71 (0.63-0.78)	0.63	Ref		Ref		Ref		Ref		Ref		Ref		Ref	
	Intermediate risk	1218/166589	0.82 (0.77-0.87)	0.73	1.16 (1.03-1.31)	0.0121	1.18 (1.05-1.33)	0.0056	1.17 (1.01-1.35)	0.0322	1.22 (1.02-1.47)	0.0292	1.22 (1.01-1.46)	0.0347	1.19 (0.99-1.43)	0.0571	1.20 (0.99-1.43)	0.0548
	High risk	426/52348	0.91 (0.83-1.00)	0.81	1.30 (1.13-1.49)	0.0003	1.32 (1.15-1.52)	0.0001	1.27 (1.07-1.51)	0.0057	1.33 (1.08-1.66)	0.0086	1.31 (1.05-1.62)	0.0163	1.25 (1.01-1.55)	0.0414	1.25 (1.01-1.56)	0.0399
	Very high risk	40/2736	1.65 (1.18-2.24)	1.46	2.34 (1.69-3.25)	<0.0001	2.38 (1.71-3.30)	<0.0001	2.25 (1.53-3.31)	<0.0001	2.46 (1.54-3.92)	0.0017	2.41 (1.50-3.85)	0.0003	2.19 (1.37-3.51)	0.001	2.17 (1.36-3.48)	0.0012

Model 1: Age + sex + genotyping array + first ten PC

Model 2: Model 1 + systolic BP + diastolic BP + BMI + waist circumference + current smoking + alcohol frequency + physical activity + eating habit

Model 3: Model 2 + HbA1c + total cholesterol + triglyceride + HDL cholesterol + LDL cholesterol + lipoprotein (a) + estimated GFR + AST + ALT + Total bilirubin + albumin + white blood cell + hemoglobin + platelet + C-reactive protein

Model 4: Model 3 + PRS-specific disease

Model 5: Model 3 + hypertension + dyslipidemia + Charlson comorbidity index

Model 6: Model 4 + aspirin + lipid-lowering agent + anti-hypertensive agent

CAD, coronary artery disease; PRS polygenic risk score; T2DM, type 2 diabetes; PC, principal component; BP, blood pressure; GFR, glomerular filtration rate; AST, Aspartate aminotransferase; ALT, Alanine aminotransferase; HR, hazard ratio; CI, confidence interval.

CAD PRS and T2DM PRS of Model 4 were adjusted for CAD and T2DM, respectively.

Supplementary Table 9. Hazard ratios and 95% confidential intervals for cardiovascular mortality according to genetic risk for CAD and lifestyle behaviors.

		No. of Events/ Total No.	Incidence/ 1000 person-year (95% CI)	Absolute risk (%)	Cardiovascular mortality					
					**Crude**		**Model 1**		**Model 2**	
					**HR (95% CI)**	***P* value**	**HR (95% CI)**	***P* value**	**HR (95% CI)**	***P* value**
**Low CAD genetic risk**	**Favorable lifestyle**	181/39836	0.51 (0.44–0.59)	0.45	Ref		Ref		Ref	
	**Intermediate lifestyle**	164/24211	0.76 (0.65–0.89)	0.68	1.49 (1.20–1.84)	<0.001	1.49 (1.21–1.84)	<0.001	1.44 (1.10–1.88)	<0.001
	**Unfavorable lifestyle**	115/8697	1.48 (1.23–1.78)	1.32	2.90 (2.30–3.67)	<0.001	2.91 (2.30–3.67)	<0.001	2.04 (1.50–2.77)	<0.001
**Intermediate CAD genetic risk**	**Favorable lifestyle**	627/117694	0.59 (0.55–0.65)	0.53	1.17 (0.99–1.38)	0.06	1.16 (0.99–1.37)	0.07	1.23 (0.99–1.53)	0.05
	**Intermediate lifestyle**	701/73788	1.07 (0.99–1.15)	0.95	2.09 (1.77–2.46)	<0.001	2.11 (1.79–2.49)	<0.001	1.79 (1.45–2.22)	<0.001
	**Unfavorable lifestyle**	420/26495	1.78 (1.61–1.96)	1.59	3.49 (2.93–4.15)	<0.001	3.58 (3.01–4.27)	<0.001	2.43 (1.93–3.06)	<0.001
**High CAD genetic risk**	**Favorable lifestyle**	244/36999	0.74 (0.65–0.84)	0.66	1.45 (1.20–1.76)	<0.001	1.46 (1.20–1.77)	<0.001	1.52 (1.19–1.95)	<0.001
	**Intermediate lifestyle**	247/23431	1.18 (1.04–1.34)	1.05	2.32 (1.91–2.81)	<0.001	2.38 (1.97–2.89)	<0.001	1.91 (1.49–2.45)	<0.001
	**Unfavorable lifestyle**	170/8675	2.20 (1.88–2.26)	1.96	4.30 (3.49–5.30)	<0.001	4.55 (3.69–5.61)	<0.001	2.77 (2.10–3.67)	<0.001
**Very high CAD genetic risk**	**Favorable lifestyle**	24/1921	1.41 (0.90–2.09)	1.25	2.75 (1.80–4.22)	<0.001	2.82 (1.84–4.31)	<0.001	2.27 (1.18–4.35)	0.01
	**Intermediate lifestyle**	22/1265	1.85 (1.22–2.96)	1.74	3.82 (2.46–5.95)	<0.001	4.14 (2.66–6.44)	<0.001	2.13 (1.11–4.09)	0.02
	**Unfavorable lifestyle**	18/466	4.38 (2.60–6.92)	3.86	8.59 (5.29–13.94)	<0.001	8.31 (5.12–13.49)	<0.001	5.55 (2.80–11.01)	<0.001

Model 1: Age + sex + genotyping array + first ten PC

Model 2: Age + sex + genotyping array + first ten PC + systolic BP + diastolic BP + BMI + waist circumference + current smoking + alcohol frequency + physical activity + eating habit + HbA1c + total cholesterol + triglyceride + HDL cholesterol + LDL cholesterol + lipoprotein (a) + estimated GFR + AST + ALT + Total bilirubin + albumin + white blood cell + hemoglobin + platelet + C-reactive protein + PRS specific disease + hypertension + dyslipidemia + heart failure + cancer + chronic kidney disease + stroke + chronic lung disease + chronic liver disease + Charlson comorbidity index + aspirin + lipid-lowering agent + anti-hypertensive agent

CAD, coronary artery disease; PRS polygenic risk score; PC, principal component; BP, blood pressure; GFR, glomerular filtration rate; AST, Aspartate aminotransferase; ALT, Alanine aminotransferase; HR, hazard ratio; CI, confidence interval.

Supplementary Table 10. Hazard ratios and 95% confidential intervals for cardiovascular mortality according to genetic risk for T2DM and lifestyle behaviors.

		No. of Events/ Total No.	Incidence/ 1000 person-year (95% CI)	Absolute risk (%)	Cardiovascular mortality					
					Crude		Model 1		Model 2	
					HR (95% CI)	*P* value	HR (95% CI)	*P* value	HR (95% CI)	*P* value
**Low T2DM genetic risk**	**Favorable lifestyle**	194/40929	0.53 (0.50–0.61)	0.47	Ref		Ref		Ref	
	**Intermediate lifestyle**	194/24001	0.91 (0.78–1.04)	0.80	1.70 (1.40–2.08)	<0.001	1.72 (1.41–2.10)	<0.001	1.43 (1.10–1.84)	0.006
	**Unfavorable lifestyle**	107/7855	1.53 (1.25–1.84)	1.36	2.86 (2.26–3.62)	<0.001	2.90 (2.29–3.68)	<0.001	1.77 (1.29–2.42)	<0.001
**Intermediate T2DM genetic risk**	**Favorable lifestyle**	642/117806	0.61 (0.57–0.66)	0.54	1.15 (0.98–1.35)	0.09	1.16 (0.99–1.37)	0.06	1.19 (0.97–1.46)	0.09
	**Intermediate lifestyle**	678/73666	1.03 (0.96–1.11)	0.92	1.94 (1.66–2.28)	<0.001	1.99 (1.70–2.34)	<0.001	1.64 (1.34–2.02)	<0.001
	**Unfavorable lifestyle**	443/26649	1.87 (1.70–2.05)	1.66	3.51 (2.96–4.15)	<0.001	3.63 (3.07–4.30)	<0.001	2.35 (1.89–2.93)	<0.001
**High T2DM genetic risk**	**Favorable lifestyle**	227/35957	0.71 (0.62–0.81)	0.63	1.34 (1.10–1.62)	0.003	1.35 (1.11–1.63)	0.002	1.28 (1.01–1.63)	0.04
	**Intermediate lifestyle**	237/23718	1.12 (0.98–1.27)	1.00	2.11 (1.75–2.55)	<0.001	2.18 (1.80–2.64)	<0.001	1.76 (1.39–2.24)	<0.001
	**Unfavorable lifestyle**	159/9291	1.93 (1.64–2.25)	1.71	3.62 (2.94–4.47)	<0.001	3.87 (3.14–4.77)	<0.001	2.43 (1.85–3.20)	<0.001
**Very high T2DM genetic risk**	**Favorable lifestyle**	13/1758	0.83 (0.44–1.43)	0.74	1.57 (0.90–2.76)	0.11	1.59 (0.90–2.78)	0.11	1.16 (0.54–2.50)	0.70
	**Intermediate lifestyle**	25/1310	2.15 (1.39–3.18)	1.91	4.05 (2.67–6.14)	<0.001	4.40 (2.90–6.67)	<0.001	2.49 (1.40–4.42)	0.03
	**Unfavorable lifestyle**	14/538	2.94 (1.61–4.93)	2.60	5.53 (3.21–9.50)	<0.001	5.84 (3.39–10.04)	<0.001	4.34 (2.27–8.30)	<0.001

Model 1: Age + sex + genotyping array + first ten PC

Model 2: Age + sex + genotyping array + first ten PC + systolic BP + diastolic BP + BMI + waist circumference + current smoking + alcohol frequency + physical activity + eating habit
+ HbA1c + total cholesterol + triglyceride + HDL cholesterol + LDL cholesterol + lipoprotein (a) + estimated GFR + AST + ALT + Total bilirubin + albumin + white blood cell + hemoglobin + platelet + C-reactive protein
+ PRS specific disease + hypertension + dyslipidemia + heart failure + cancer + chronic kidney disease + stroke + chronic lung disease + chronic liver disease + Charlson comorbidity index
+ aspirin + lipid-lowering agent + anti-hypertensive agent

T2DM, type 2 diabetes mellitus; PRS polygenic risk score; PC, principal component; BP, blood pressure; GFR, glomerular filtration rate; AST, Aspartate aminotransferase; ALT, Alanine aminotransferase; HR, hazard ratio; CI, confidence interval.

Supplementary Table 11. Hazard ratios and 95% confidential intervals for cardiovascular mortality according to genetic risk and age category.

CV mortality		Age 40-54									Age 55-69								
		No. of Events/ Total No.	Incidence/ 1000 person-year	Absolute risk (%)	Crude		Model 1		Model 2		No. of Events/ Total No.	Incidence/ 1000 person-year	Absolute risk (%)	Crude		Model 2		Model 2	
					HR (95% CI)	*P* value	HR (95% CI)	*P* value	HR (95% CI)	*P* value				HR (95% CI)	*P* value	HR (95% CI)	*P* value	HR (95% CI)	*P* value
CAD PRS	Low risk	70/28502	0.27 (0.21–0.35)	0.25	Ref		Ref		Ref		446/47087	1.07 (0.97–1.17)	0.95	Ref		Ref		Ref	
	Intermediate risk	220/85868	0.29 (0.25–0.33)	0.26	1.17 (0.86–1.58)	0.32	1.06 (0.81–1.39)	0.67	0.89 (0.59–1.35)	0.58	1686/140899	1.35 (1.28–1.41)	1.20	1.24 (1.12–1.37)	<0.001	1.26 (1.13–1.40)	0.000	1.19 (1.02–1.40)	0.03
	High risk	110/27425	0.45 (0.37–0.54)	0.40	1.81 (1.30–2.54)	0.0004	1.64 (1.22–2.22)	0.001	1.50 (0.95–2.38)	0.08	611/44351	1.55 (1.43–1.68)	1.38	1.44 (1.28–1.62)	<0.001	1.47 (1.30–1.66)	0.000	1.34 (1.11–1.62)	0.002
	Very high risk	10/1451	0.77 (0.37–1.41)	0.69	2.96 (1.41–6.21)	0.004	2.85 (1.47–5.53)	0.002	2.15 (0.64–7.23)	0.22	57/2326	2.78 (2.10–3.60)	2.45	2.57 (1.96–3.37)	<0.001	2.63 (2.00–3.47)	0.000	1.90 (1.14–3.19)	0.01
T2DM PRS	Low risk	58/28336	0.23 (0.17–0.30)	0.20	Ref		Ref		Ref		496/47271	1.18 (1.08–1.29)	1.05	Ref		Ref		Ref	
	Intermediate risk	258/86208	0.33 (0.29–0.38)	0.30	1.66 (1.21–2.29)	0.002	1.52 (1.14–2.01)	0.004	1.57 (0.98–2.52)	0.06	1658/140575	1.33 (1.27–1.39)	1.18	1.11 (1.00–1.22)	0.05	1.12 (1.01–1.23)	0.02	1.11 (0.95–1.29)	0.21
	High risk	87/27257	0.36 (0.29–0.44)	0.32	1.67 (1.15–2.42)	0.007	1.62 (1.16–2.26)	0.005	1.46 (0.84–2.52)	0.18	596/44487	1.51 (1.39–1.64)	1.34	1.28 (1.13–1.43)	<0.001	1.30 (1.15–1.46)	0.000	1.23 (1.02–1.48)	0.03
	Very high risk	7/1445	0.54 (0.22–1.12)	0.48	1.79 (0.64–4.96)	0.27	2.46 (1.12–5.40)	0.02	0.57 (0.08–4.33)	0.59	50/2330	2.43 (1.80–3.21)	2.15	2.10 (1.58–2.78)	<0.001	2.10 (1.57–2.81)	0.000	1.96 (1.28–3.00)	0.002

Model 1: Age + sex + genotyping array + first ten PC

Model 2: Age + sex + genotyping array + first ten PC + systolic BP + diastolic BP + BMI + waist circumference + current smoking + alcohol frequency + physical activity + eating habit
+ HbA1c + total cholesterol + triglyceride + HDL cholesterol + LDL cholesterol + lipoprotein (a) + estimated GFR + AST + ALT + Total bilirubin + albumin + white blood cell + hemoglobin + platelet + C-reactive protein
+ PRS specific disease + hypertension + dyslipidemia + heart failure + cancer + chronic kidney disease + stroke + chronic lung disease + chronic liver disease + Charlson comorbidity index
+ aspirin + lipid-lowering agent + anti-hypertensive agent

T2DM, type 2 diabetes mellitus; PRS polygenic risk score; PC, principal component; BP, blood pressure; GFR, glomerular filtration rate; AST, Aspartate aminotransferase; ALT, Alanine aminotransferase; HR, hazard ratio; CI, confidence interval.

Supplementary Table 12. Hazard ratios and 95% confidential intervals for the cardiovascular mortality according to genetic risk and sex category.

CV mortality		Men									Women								
		No. of Events/ Total No.	Incidence/ 1000 person-year	Absolute risk (%)	Crude		Model 1		Model 2		No. of Events/ Total No.	Incidence/ 1000 person-year	Absolute risk (%)	Crude		Model 1		Model 2	
					HR (95% CI)	*P* value	HR (95% CI)	*P* value	HR (95% CI)	*P* value				HR (95% CI)	*P* value	HR (95% CI)	*P* value	HR (95% CI)	*P* value
**CAD PRS**	Low risk	362/35020	1.16 (1.05–1.29)	1.03	Ref		Ref		Ref		154/40569	0.43 (0.36–0.50)	0.38	Ref		Ref		Ref	
	Intermediate risk	1400/104899	1.50 (1.42–1.58)	1.33	1.29 (1.15–1.45)	<0.001	1.29 (1.15–1.45)	<0.001	1.22 (1.03–1.45)	0.02	505/121809	0.46 (0.42–0.51)	0.41	1.10 (0.91–1.31)	0.32	1.10 (0.92–1.32)	0.31	1.01 (0.74–1.37)	0.95
	High risk	536/33111	1.82 (1.67–1.98)	1.62	1.57 (1.37–1.79)	<0.001	1.59 (1.39–1.81)	<0.001	1.47 (1.20–1.79)	<0.001	185/38665	0.54 (0.46–0.61)	0.48	1.26 (1.02–1.56)	0.03	1.27 (1.02–1.57)	0.03	1.13 (0.78–1.63)	0.53
	Very high risk	50/1740	3.24 (2.41–4.28)	2.87	2.78 (2.07–3.73)	<0.001	2.85 (2.12–3.83)	<0.001	2.33 (1.42–3.82)	<0.001	17/2037	0.94 (0.55–1.50)	0.83	2.22 (1.34–3.66)	0.002	2.27 (1.37–3.74)	0.001	0.50 (0.07–3.66)	0.50
**T2DM PRS**	Low risk	397/34867	1.28 (1.16–1.41)	1.14	Ref		Ref		Ref		157/40740	0.43 (0.37–0.50)	0.39	Ref		Ref		Ref	
	Intermediate risk	1407/104888	1.51 (1.43–1.59)	1.34	1.18 (1.06–1.32)	0.004	1.20 (1.08–1.35)	0.001	1.21 (1.02–1.43)	0.03	507/121829	0.47 (0.43–0.51)	0.42	1.08 (0.90–1.29)	0.43	1.09 (0.91–1.30)	0.36	1.03 (0.75–1.42)	0.83
	High risk	500/33238	1.70 (1.55–1.85)	1.50	1.33 (1.17–1.52)	<0.001	1.37 (1.20–1.56)	<0.001	1.25 (1.02–1.53)	0.03	183/38506	0.53 (0.46–0.62)	0.48	1.24 (0.99–1.53)	0.05	1.25 (1.01–1.55)	0.04	1.33 (0.92–1.91)	0.13
	Very high risk	43/1770	2.75 (1.99–3.70)	2.43	2.13 (1.55–2.92)	<0.001	2.22 (1.61–3.05)	<0.001	1.92 (1.22–3.04)	0.005	14/2005	0.78 (0.43–1.32)	0.70	1.79 (1.04–3.10)	0.04	1.90 (1.10–3.28)	0.02	1.54 (0.55–4.29)	0.41

Model 1: Age + sex + genotyping array + first ten PC

Model 2: Age + sex + genotyping array + first ten PC + systolic BP + diastolic BP + BMI + waist circumference + current smoking + alcohol frequency + physical activity + eating habit

+ HbA1c + total cholesterol + triglyceride + HDL cholesterol + LDL cholesterol + lipoprotein (a) + estimated GFR + AST + ALT + Total bilirubin + albumin + white blood cell + hemoglobin + platelet + C-reactive protein

+ PRS specific disease + hypertension + dyslipidemia + heart failure + cancer + chronic kidney disease + stroke + chronic lung disease + chronic liver disease + Charlson comorbidity index

+ aspirin + lipid-lowering agent + anti-hypertensive agent

T2DM, type 2 diabetes mellitus; PRS polygenic risk score; PC, principal component; BP, blood pressure; GFR, glomerular filtration rate; AST, Aspartate aminotransferase; ALT, Alanine aminotransferase; HR, hazard ratio; CI, confidence interval.

Supplementary Table 13. Hazard ratios and 95% confidential intervals for cardiovascular mortality according to genetic risk and prevalent disease of T2DM or CAD at baseline.

CV mortality		T2DM at baseline									CAD at baseline								
		No. of Events/ Total No.	Incidence/ 1000 person-year	Absolute mortality rate	Crude		Model 1		Model 2		No. of Events/ Total No.	Incidence/ 1000 person-year	Absolute mortality rate	Crude		Model 1		Model 2	
					HR (95% CI)	*P* value	HR (95% CI)	*P* value	HR (95% CI)	*P* value				HR (95% CI)	*P* value	HR (95% CI)	*P* value	HR (95% CI)	*P* value
CAD PRS	Low risk	70/2725	2.91 (2.26–3.67)	2.57	Ref		Ref		Ref		120/2950	4.62 (3.83–5.53)	4.07	Ref		Ref		Ref	
	Intermediate risk	266/9030	3.34 (2.95–3.77)	2.95	1.22 (0.94–1.57)	0.14	1.23 (0.95–1.59)	0.12	0.88 (0.59–1.32)	0.55	547/12592	4.95 (4.54–5.39)	4.34	1.14 (0.96–1.35)	0.14	1.11 (0.94–1.32)	0.23	1.19 (0.90–1.58)	0.22
	High risk	120/3099	4.40 (3.65–5.26)	3.87	1.65 (1.23–2.21)	0.0008	1.71 (1.28–2.29)	0.0003	1.14 (0.71–1.84)	0.58	247/5639	5.00 (4.40–5.67)	4.38	1.16 (0.94–1.43)	0.15	1.15 (0.93–1.42)	0.19	1.43 (1.02–2.01)	0.04
	Very high risk	14/180	8.94 (4.89–15.00)	7.78	3.60 (1.99–6.48)	<0.001	3.86 (2.14–6.98)	<0.001	4.16 (1.56–11.10)	0.004	24/497	5.51 (3.53–8.20)	4.83	1.19 (0.63–2.24)	0.60	1.19 (0.63–2.25)	0.59	0.66 (0.09–4.83)	0.69
T2DM PRS	Low risk	50/1758	3.22 (2.39–4.25)	2.84	Ref		Ref		Ref		142/3675	4.39 (3.70–5.17)	3.86	Ref		Ref		Ref	
	Intermediate risk	264/8724	3.43 (3.03–3.87)	3.03	1.16 (0.91–1.49)	0.23	1.20 (0.94–1.54)	0.14	1.34 (0.88–2.04)	0.18	559/13183	4.83 (4.44–5.25)	4.24	1.19 (0.99–1.41)	0.06	1.20 (1.01–1.43)	0.04	1.25 (0.93–1.67)	0.14
	High risk	138/4222	3.71 (3.12–4.38)	3.27	1.19 (0.88–1.60)	0.26	1.23 (0.92–1.66)	0.17	1.16 (0.69–1.95)	0.58	211/4529	5.32 (4.63–6.10)	4.66	1.35 (1.09–1.66)	0.005	1.37 (1.11–1.69)	0.003	1.37 (0.97–1.93)	0.08
	Very high risk	18/330	6.23 (3.69–9.85)	5.45	2.67 (1.42–5.01)	0.002	2.99 (1.59–5.62)	0.0006	2.82 (1.03–7.72)	0.04	26/291	10.42 (6.81–15.27)	8.93	2.19 (1.33–3.61)	0.002	2.31 (1.40–3.80)	0.001	2.46 (1.11–5.46)	0.03

Model 1: Age + sex + genotyping array + first ten PC

Model 2: Age + sex + genotyping array + first ten PC + systolic BP + diastolic BP + BMI + waist circumference + current smoking + alcohol frequency + physical activity + eating habit + HbA1c + total cholesterol + triglyceride + HDL cholesterol + LDL cholesterol + lipoprotein (a) + estimated GFR + AST + ALT + Total bilirubin + albumin + white blood cell + hemoglobin + platelet + C-reactive protein + PRS specific disease + hypertension + dyslipidemia + heart failure + cancer + chronic kidney disease + stroke + chronic lung disease + chronic liver disease + Charlson comorbidity index + aspirin + lipid-lowering agent + anti-hypertensive agent

T2DM, type 2 diabetes mellitus; PRS polygenic risk score; PC, principal component; BP, blood pressure; GFR, glomerular filtration rate; AST, Aspartate aminotransferase; ALT, Alanine aminotransferase; HR, hazard ratio; CI, confidence interval.

Supplementary Figure 1. Density and prevalence plot according to the PRS distribution (A) CAD and (B) T2DM.

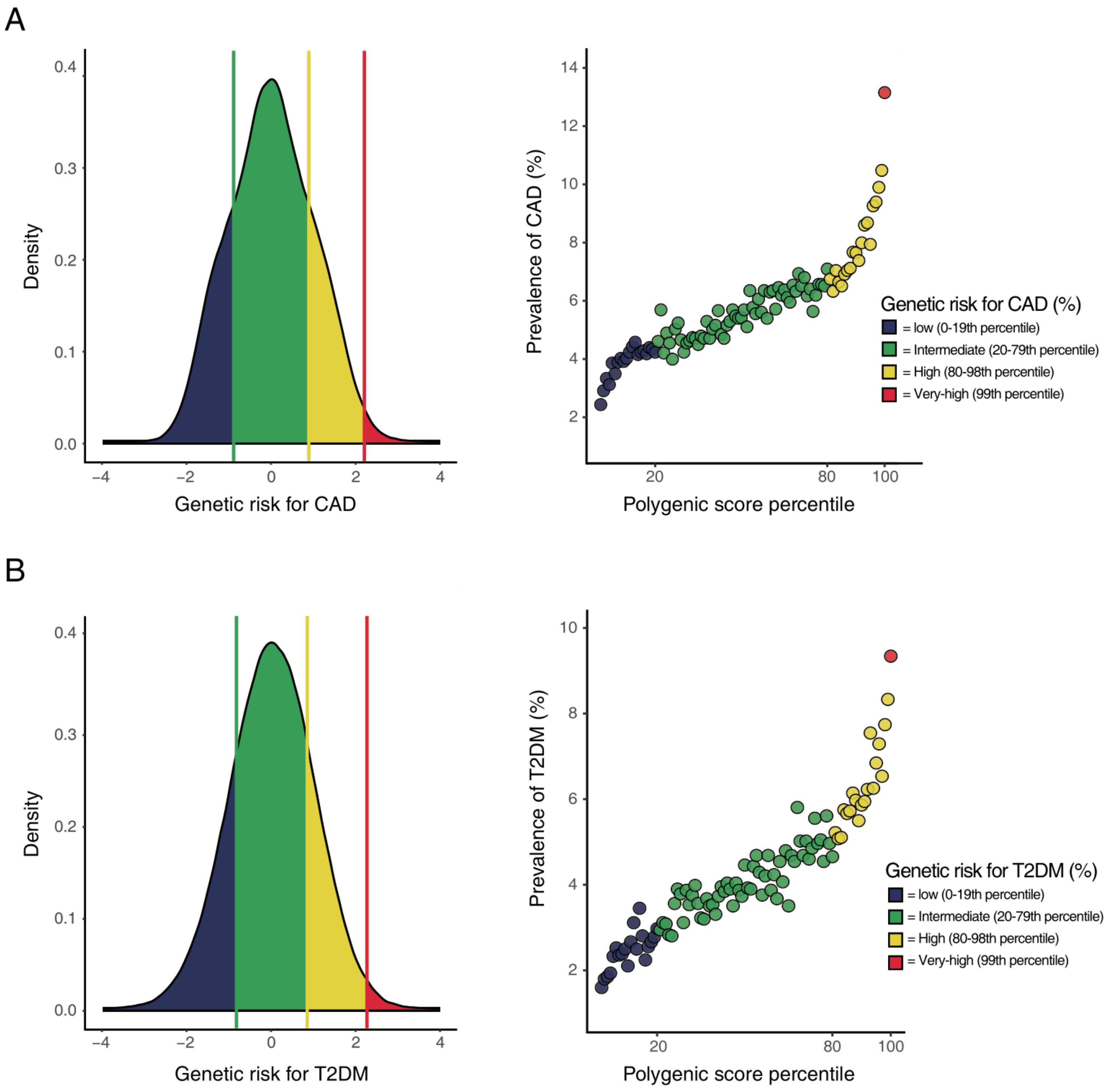

PRS, polygenic risk score; CAD, coronary artery disease; T2DM, type 2 diabetes mellitus.

Supplementary Figure 2. 10-year cardiovascular mortality rates according to (A) genetic risk for CAD and (B) genetic risk for T2DM and lifestyle behavior.

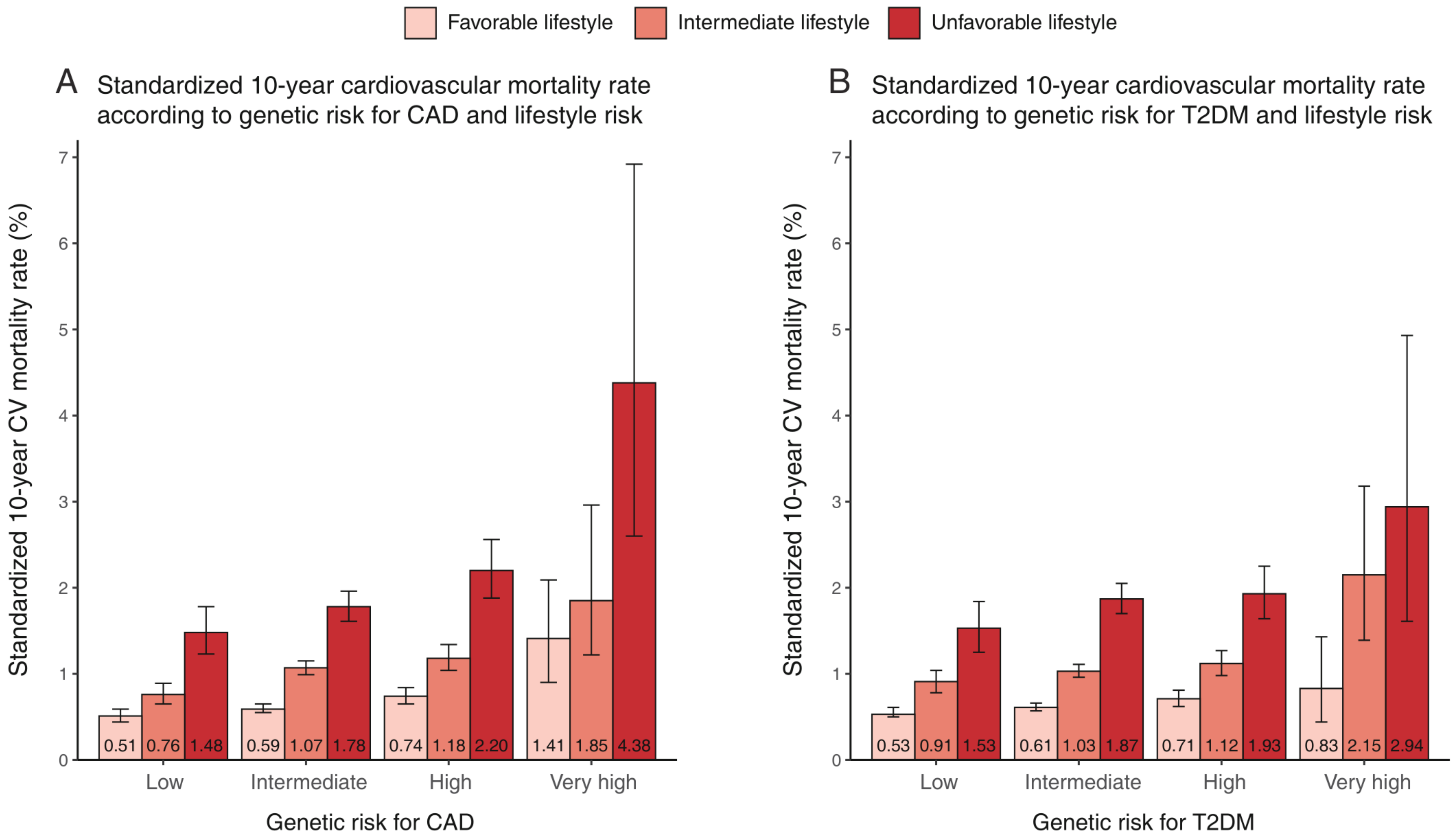

CV, cardiovascular; CAD, coronary artery disease; T2DM, type 2 diabetes mellitus.

Supplementary Figure 3. Forest plot of cardiovascular mortality according to genetic risk for CAD and four lifestyle behaviors.

Subgroup		No. of Event /Total No.	Incidence /1000 person-yr	Absolute risk (%)	Adjusted HR (95% CI)	*P* value
**Genetic risk for CAD**						
Low genetic risk	No current smoker	395/67943	0.65 (0.59−0.72)	0.58	Ref	
	Current smoker	121/7646	1.78 (1.47−2.12)	1.58	2.86 (2.33−3.51)	<0.001
Intermediate genetic risk	No current smoker	1478/203196	0.82 (0.78−0.86)	0.73	1.25 (1.12−1.40)	<0.001
	Current smoker	428/23571	2.04 (1.85−2.24)	1.82	3.33 (2.90−3.82)	<0.001
High genetic risk	No current smoker	549/64201	0.96 (0.88−1.04)	0.86	1.49 (1.31−1.70)	<0.001
	Current smoker	172/7575	2.55 (2.18−2.96)	2.27	4.26 (3.56−5.09)	<0.001
Very high genetic risk	No current smoker	51/3426	1.68 (1.25−2.20)	1.49	2.66 (1.98−3.56)	<0.001
	Current smoker	16/351	5.14 (2.94−8.36)	4.56	8.27 (5.02−13.64)	<0.001
**Genetic risk for CAD**						
Low genetic risk	No obesity	314/57575	0.61 (0.55−0.68)	0.55	Ref	
	Obesity	202/18014	1.26 (1.09−1.45)	1.12	1.93 (1.62−2.30)	<0.001
Intermediate genetic risk	No obesity	1175/171215	0.77 (0.73−0.82)	0.69	1.25 (1.11−1.42)	<0.001
	Obesity	731/55552	1.48 (1.37−1.59)	1.32	2.31 (2.02−2.64)	<0.001
High genetic risk	No obesity	439/53704	0.92 (0.83−1.01)	0.82	1.51 (1.31−1.75)	<0.001
	Obesity	282/18072	1.76 (1.56−1.97)	1.56	2.79 (2.37−3.27)	<0.001
Very high genetic risk	No obesity	40/2761	1.63 (1.16−2.22)	1.45	2.70 (1.94−3.75)	<0.001
	Obesity	27/1016	3.01 (1.98−4.38)	2.66	4.96 (3.35−7.35)	<0.001
**Genetic risk for CAD**						
Low genetic risk	Regular physical activity	298/52273	0.64 (0.57−0.72)	0.57	Ref	
	Inactivity	218/23316	1.05 (0.91−1.20)	0.93	1.61 (1.35−1.92)	<0.001
Intermediate genetic risk	Regular physical activity	1055/156215	0.76 (0.71−0.81)	0.68	1.19 (1.04−1.35)	0.009
	Inactivity	851/70552	1.35 (1.26−1.45)	1.21	2.08 (1.82−2.38)	<0.001
High genetic risk	Regular physical activity	417/49341	0.95 (0.86−1.05)	0.85	1.50 (1.29−1.74)	<0.001
	Inactivity	304/22435	1.52 (1.35−1.70)	1.36	2.38 (2.03−2.80)	<0.001
Very high genetic risk	Regular physical activity	35/2547	1.55 (1.08−2.15)	1.37	2.54 (1.79−3.61)	<0.001
	Inactivity	32/1230	2.93 (2.00−4.13)	2.60	4.43 (3.08−6.38)	<0.001
**Genetic risk for CAD**						
Low genetic risk	Ideal eating habit	453/67919	0.75 (0.68−0.82)	0.67	Ref	
	Poor eating habit	63/7670	0.92 (0.71−1.18)	0.82	1.12 (0.93−1.35)	0.24
Intermediate genetic risk	Ideal eating habit	1662/203075	0.92 (0.88−0.96)	0.82	1.19 (0.99−1.42)	0.05
	Poor eating habit	244/23692	1.15 (1.01−1.31)	1.03	1.40 (1.19−1.65)	<0.001
High genetic risk	Ideal eating habit	624/64299	1.09 (1.01−1.18)	0.97	1.45 (1.18−1.78)	<0.001
	Poor eating habit	97/7477	1.45 (1.18−1.77)	1.30	1.70 (1.42−2.03)	<0.001
Very high genetic risk	Ideal eating habit	58/3394	1.92 (1.46−2.49)	1.71	2.61 (1.62−4.21)	<0.001
	Poor eating habit	9/383	2.65 (1.21−5.04)	2.35	3.03 (2.19−4.18)	<0.001

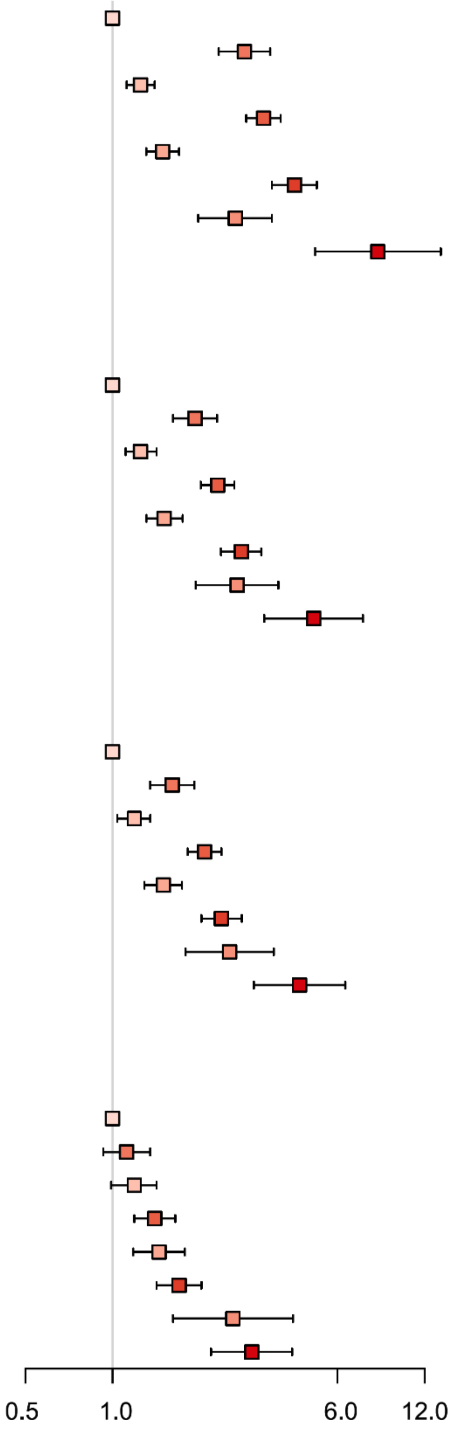

CAD, coronary artery disease; HR, hazard ratio; CI, confidence interval.

Supplementary Figure 4. Forest plot of cardiovascular mortality according to genetic risk for T2DM and four lifestyle behaviors.

Subgroup		No. of Event /Total No.	Incidence /1000 person-yr	Absolute risk (%)	Adjusted HR (95% CI)	P value
**Genetic risk for T2DM**						
Low genetic risk	No current smoker	447/67993	0.74 (0.67−0.81)	0.66	Ref	
	Current smoker	107/7614	1.57 (1.29−1.90)	1.41	2.25 (1.82−2.78)	<0.001
Intermediate genetic risk	No current smoker	1460/203303	0.81 (0.77−0.85)	0.72	1.11 (0.99−1.23)	0.06
	Current smoker	456/23480	2.18 (1.98−2.39)	1.94	3.18 (2.79−3.62)	<0.001
High genetic risk	No current smoker	519/64097	0.90 (0.83−0.99)	0.81	1.26 (1.11−1.43)	<0.001
	Current smoker	164/7647	2.41 (2.06−2.81)	2.14	3.61 (3.02−4.32)	<0.001
Very high genetic risk	No current smoker	47/3373	1.57 (1.16−2.09)	1.39	2.21 (1.63−2.98)	<0.001
	Current smoker	10/402	2.79 (1.34−5.13)	2.49	4.33 (2.31−8.10)	<0.001
**Genetic risk for T2DM**						
Low genetic risk	No obesity	357/59366	0.67 (0.61−0.75)	0.60	Ref	
	Obesity	197/16241	1.36 (1.18−1.57)	1.21	1.92 (1.61−2.29)	<0.001
Intermediate genetic risk	No obesity	1174/171261	0.77 (0.73−0.81)	0.69	1.16 (1.03−1.31)	0.01
	Obesity	742/55522	1.50 (1.40−1.61)	1.34	2.16 (1.90−2.45)	<0.001
High genetic risk	No obesity	407/52057	0.88 (0.79−0.97)	0.78	1.33 (1.15−1.53)	<0.001
	Obesity	276/19687	1.58 (1.40−1.78)	1.40	2.30 (1.97−2.70)	<0.001
Very high genetic risk	No obesity	30/2571	1.31 (0.89−1.88)	1.17	2.00 (1.38−2.90)	<0.001
	Obesity	27/1204	2.53 (1.67−3.68)	2.24	3.88 (2.62−5.74)	<0.001
**Genetic risk for T2DM**						
Low genetic risk	Regular physical activity	315/52731	0.67 (0.60−0.75)	0.60	Ref	
	Inactivity	239/22876	1.17 (1.03−1.33)	1.04	1.70 (1.43−2.01)	<0.001
Intermediate genetic risk	Regular physical activity	1082/156056	0.78 (0.73−0.83)	0.69	1.17 (1.04−1.33)	0.01
	Inactivity	834/70727	1.32 (1.23−1.41)	1.18	1.97 (1.73−2.24)	<0.001
High genetic risk	Regular physical activity	378/49044	0.87 (0.78−0.96)	0.77	1.31 (1.13−1.53)	<0.001
	Inactivity	305/22700	1.51 (1.34−1.69)	1.34	2.26 (1.93−2.65)	<0.001
Very high genetic risk	Regular physical activity	30/2545	1.33 (0.90−1.90)	1.18	2.04 (1.40−2.97)	<0.001
	Inactivity	27/1230	2.48 (1.63−3.61)	2.20	3.81 (2.57−5.65)	<0.001
**Genetic risk for T2DM**						
Low genetic risk	Ideal eating habit	485/67749	0.80 (0.73−0.88)	0.72	Ref	
	Poor eating habit	69/7858	0.98 (0.76−1.24)	0.88	1.24 (1.03−1.49)	0.02
Intermediate genetic risk	Ideal eating habit	1674/203338	0.92 (0.88−0.97)	0.82	1.26 (1.06−1.50)	0.01
	Poor eating habit	242/23445	1.16 (1.02−1.31)	1.03	1.41 (1.20−1.66)	<0.001
High genetic risk	Ideal eating habit	589/64197	1.03 (0.95−1.12)	0.92	1.34 (1.08−1.65)	0.006
	Poor eating habit	94/7547	1.40 (1.13−1.71)	1.25	1.65 (1.38−1.97)	<0.001
Very high genetic risk	Ideal eating habit	49/3403	1.62 (1.20−2.15)	1.44	2.26 (1.37−3.73)	0.001
	Poor eating habit	8/372	2.43 (1.05−4.79)	2.15	2.62 (1.86−3.71)	<0.001

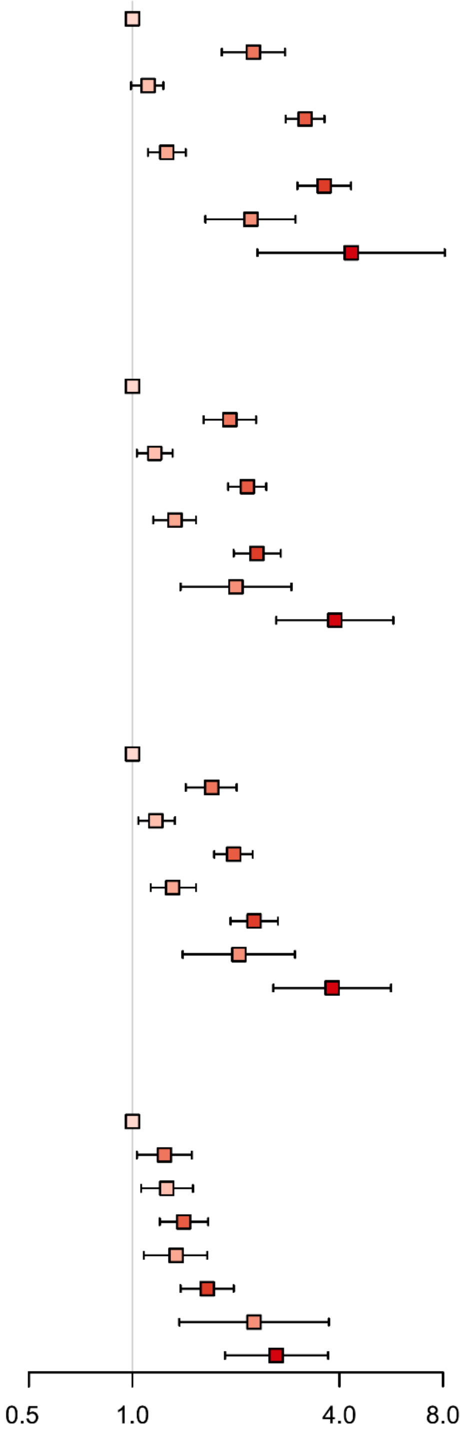

T2DM, type 2 diabetes mellitus; HR, hazard ratio; CI, confidence interval.

Supplementary Figure 5. 10-year cardiovascular mortality rates according to (A) genetic risk for CAD and age categories, (B) genetic risk for T2DM and age categories, (C) genetic risk for CAD and sex categories, and (D) genetic risk for T2DM and sex categories.

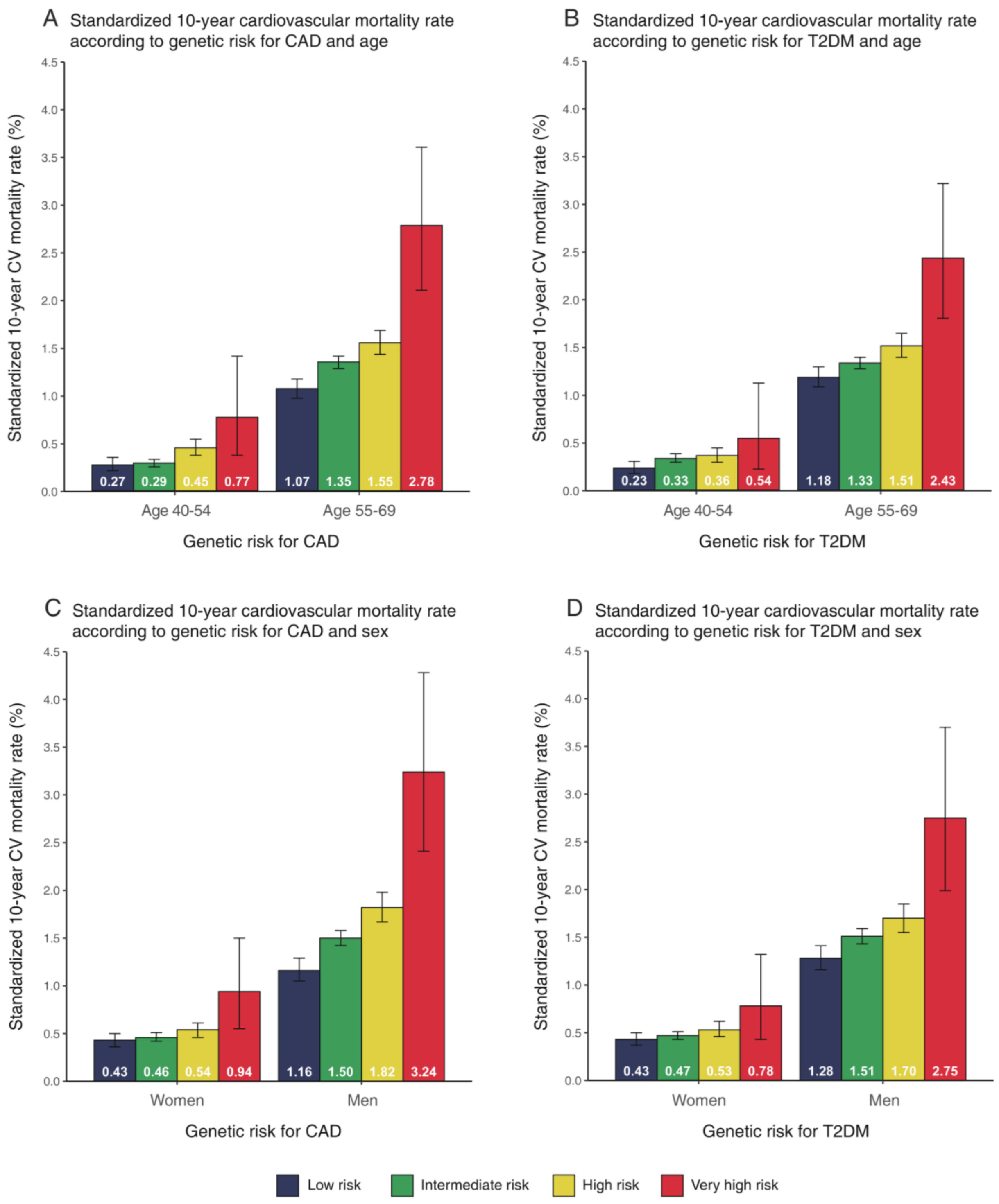

CV, cardiovascular; CAD, coronary artery disease; T2DM, type 2 diabetes mellitus.

Supplementary Figure 6. 10-year cardiovascular mortality rates according to genetic risk and prevalent disease status; (A) genetic risk for CAD and CAD history at baseline, (B) genetic risk for CAD and T2DM history at baseline, (C) genetic risk for T2DM and CAD history at baseline, and (D) genetic risk for T2DM and T2DM history at baseline.

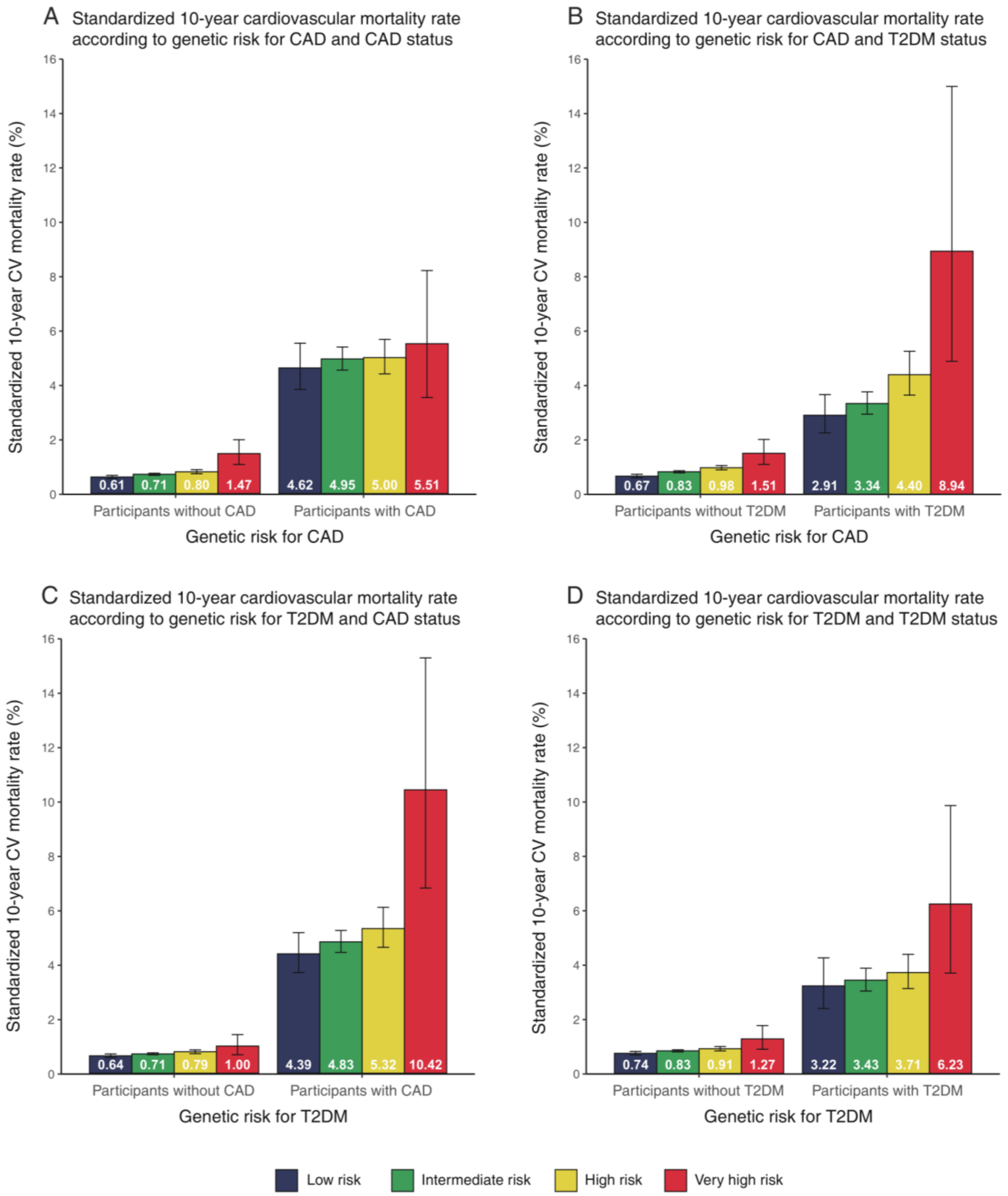

CV, cardiovascular; CAD, coronary artery disease; T2DM, type 2 diabetes mellitus.

Supplementary Figure 7. Forest plot of cardiovascular mortality according to genetic risk and sex in patients with diabetes at baseline.

Subgroup	No. of Event /Total No.	Incidence /1000 person-yr	Absolute risk (%)	Adjusted HR (95% CI)	*P* for interaction
**Genetic risk for CAD**					0.06
**Men**					
Low risk	59/1953	3.42 (2.60−4.41)	3.02	Ref	
Intermediate risk	220/5856	4.28 (3.73−4.88)	3.76	1.26 (0.95−1.68)	
High risk	92/1855	5.66 (4.56−6.94)	4.96	1.71 (1.23−2.37)	
Very high risk	8/98	9.28 (4.01−18.28)	8.16	3.07 (1.46−6.43)	
**Women**					
Low risk	15/1056	1.60 (0.90−2.64)	1.42	Ref	
Intermediate risk	48/3161	1.71 (1.26−2.27)	1.52	0.92 (0.54−1.58)	
High risk	23/1002	2.59 (1.64−3.88)	2.30	0.72 (0.35−1.51)	
Very high risk	5/53	11.06 (3.59−25.80)	9.43	2.72 (0.79−9.41)	
**Genetic risk for T2DM**					0.98
**Men**					
Low risk	68/1953	3.95 (3.07−5.01)	3.48	Ref	
Intermediate risk	226/5858	4.39 (3.84−5.00)	3.86	1.15 (0.88−1.51)	
High risk	78/1853	4.81 (3.80−6.00)	4.21	1.26 (0.91−1.75)	
Very high risk	7/98	8.13 (3.27−16.75)	7.14	2.25 (1.03−4.89)	
**Women**					
Low risk	13/1055	1.39 (0.74−2.38)	1.23	Ref	
Intermediate risk	59/3165	2.10 (1.60−2.71)	1.86	1.25 (0.68−2.31)	
High risk	15/999	1.69 (0.95−2.79)	1.50	1.43 (0.69−2.95)	
Very high risk	4/53	8.58 (2.34−21.98)	7.55	4.84 (1.35−17.30)	

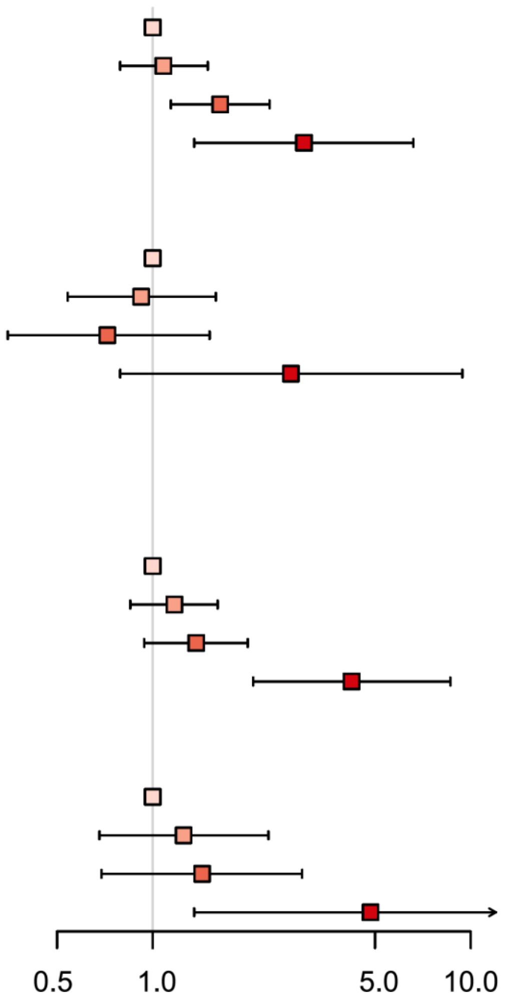

CAD, coronary artery disease; T2DM, type 2 diabetes mellitus; HR, hazard ratio; CI, confidence interval.

P values are for testing the interaction between each genetic risk category and sex.
